# What do parents and preschool staff tell us about young children's physical activity: a qualitative study

**DOI:** 10.1186/1479-5868-5-66

**Published:** 2008-12-11

**Authors:** Genevieve M Dwyer, Joy Higgs, Louise L Hardy, Louise A Baur

**Affiliations:** 1 University of Sydney Discipline of Paediatrics and Child Health, The Children's Hospital at Westmead, Locked Bag 4001 Westmead, NSW 2145, Australia; 2 The Education for Practice Institute, Charles Sturt University, 16 Masons Drive North Parramatta, NSW 2151, Australia; 3 NSW Centre for Overweight and Obesity, Level 2, K25 Medical Foundation Building University of Sydney, NSW 2006, Australia

## Abstract

**Background:**

Physical activity and small screen recreation are two modifiable behaviours associated with childhood obesity and the development of chronic health problems. Parents and preschool staff shape behaviour habits in young children. The aims of this qualitative study were to explore the attitudes, values, knowledge and understanding of parents and carers of preschool-age children in relation to physical activity and small screen recreation and to identify influences upon these behaviours.

**Methods:**

This research involved a focus group study with parents and carers of the target population. A purposive sample of 39 participants (22 parents, 17 carers) participated in 9 focus groups. Participants were drawn from three populations of interest: those from lower socioeconomic status, and Middle-Eastern and Chinese communities in the Sydney (Australia) metropolitan region.

**Results:**

All participants understood the value of physical activity and the impact of excessive small screen recreation but were unfamiliar with national guidelines for these behaviours. Participants described the nature and activity patterns of young children; however, the concept of activity 'intensity' in this age group was not a meaningful term. Factors which influenced young children's physical activity behaviour included the child's personality, the physical activity facilities available, and the perceived safety of their community. Factors facilitating physical activity included a child's preference for being active, positive parent or peer modelling, access to safe play areas, organised activities, preschool programs and a sense of social connectedness. Barriers to physical activity included safety concerns exacerbated by negative media stories, time restraints, financial constraints, cultural values favouring educational achievement, and safety regulations about equipment design and use within the preschool environment. Parents considered that young children are naturally 'programmed' to be active, and that society 'de-programs' this behaviour. Staff expressed concern that free, creative active play was being lost and that alternate activities were increasingly sedentary.

**Conclusion:**

The findings support the relevance of the socioecological model of behavioural influences to young children's physical activity. In this age group, efforts may best be directed at emphasising national guidelines for small screen recreation and educating families and carers about the importance of creative, free play to reinforce the child's inherent nature to be active.

## Background

Physical activity is a pre-requisite for optimal growth and development in children [1] and is associated with a range of health benefits [2]. Further, physical activity via play, leisure and recreational activities, provides opportunities for children to develop their sensorimotor, cognitive and socio-emotional capacities and promoting a sense of psychological well-being [3-5]. Excessive sedentariness among children potentially leads to the development of chronic health problems during adolescence and adulthood including obesity, osteoporosis, diabetes and cardiovascular diseases [6-9].

One in five preschool-age children in Australia are overweight or obese [10], but the prevalence of childhood obesity is not uniform across all sociocultural groups. In older children, those from a lower socioeconomic (SES) or a Middle-Eastern background are at higher risk of obesity [11]. Similarly there is considerable variation in the obesity-related behaviours among children. Children from Chinese ethnic backgrounds have diminished physical activity patterns outside of school hours which have been attributed to this ethnic group's societal focus on scholastic achievement [12]. For this group, the acculturation of Western lifestyle patterns following immigration to countries such as Australia or the USA [13] potentially increases the risk of developing overweight and obesity. Inactivity and increasing patterns of sedentary behaviour, particularly small screen recreation (SSR), are associated with the development of overweight and obesity, therefore the aim of this study was to identify the influences upon young children's physical activity behaviours and SSR.

### Developing a model to explore physical activity behaviour in children

According to the socioecological model described by McLeroy [14] factors which influence adult health behaviours occur within a multi-layered context, starting from within the individual to the broader social, community and organizational environments. At each level there may be facilitators and/or barriers to healthy behaviours that work in a synergistic fashion. Similar levels of influence have been reported in relation to children's physical activity and sedentary behaviour (television viewing) [15-18]

**Figure 1:**
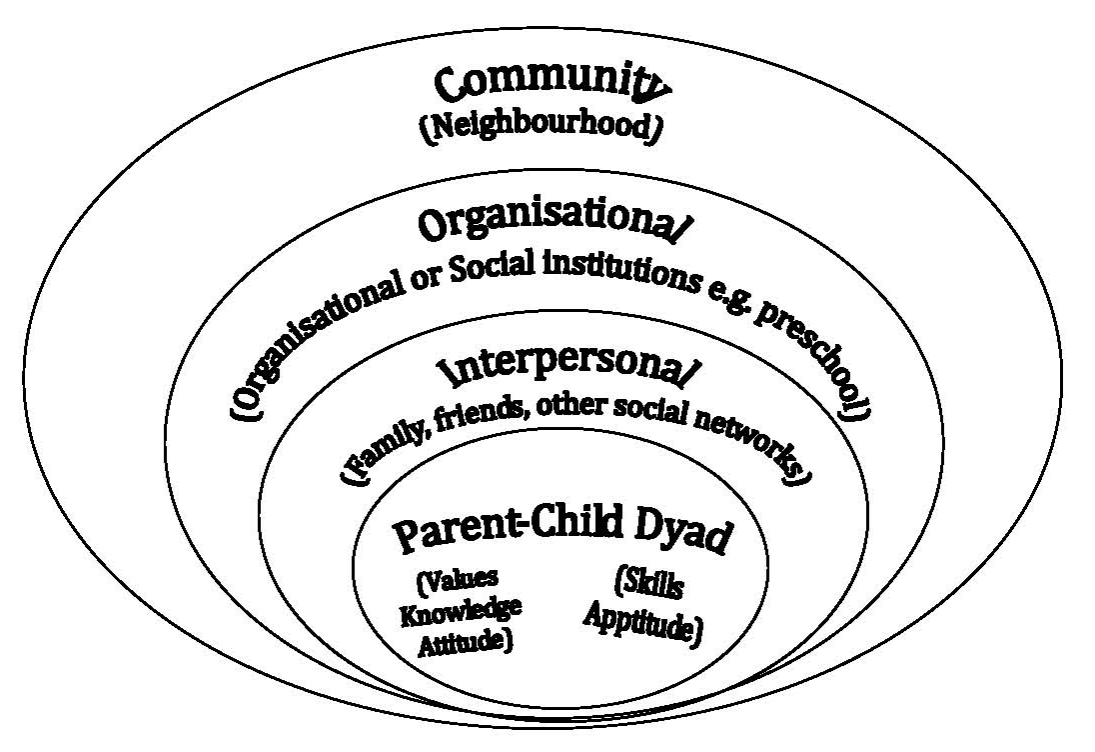
**Socioecological model of influences upon a child's physical activity behaviour (adapted from McLeroy et al**[14]).

In this study we sought to examine how well this conceptual model could be applied to understanding the influences upon physical activity behaviour in children. Figure 1 shows the McLeroy model which was modified so that the central element is the parent-child dyad, rather than the individual, because of the innate interactive influences of this dyad for children in the preschool-age group [[19,20] p337].

Specifically, this study explored the attitudes, values, knowledge and understanding of parents and carers of preschool-age children (i.e., 3–5 year olds) about physical activity and SSR and to ascertain factors which influence these behaviours.

## Methods

A qualitative focus group research design was chosen because it allowed the topic area of physical activity and SSR to be explored within the context of the participants' social setting, stage of life and experience [21].

### Participant recruitment

A purposive sampling approach was used to recruit participants living in metropolitan Sydney, Australia. Parents of children from lower SES, Middle Eastern and Chinese background were targeted because these groups are at increased risk of adopting less healthy lifestyles associated with the development of overweight and obesity [11,12]. Parents were required to be able to converse comfortably in English to be eligible for the study. This requirement assisted the focus groups to run smoothly and data collection could be managed without the need for interpreters.

Local council regions with a high proportion of low SES, Middle-Eastern and Chinese communities, were identified using the Australian Government Bureau of Statistics (ABS) Census data for 2001 and a children's service agency assisted with recruitment of preschools in these regions. The preschools' and participants' residential postcode was used as a proxy for SES, based on the ABS's Socio-Economic Indexes for Areas' (SEIFA) Index of Relative Socioeconomic Disadvantage, and was used to rank participants in tertiles of SES (low, medium or high) [22]. This index describes the socioeconomic aspects of geographical areas and includes indices on income, educational attainment, unemployment and proportion of people in unskilled occupations. The median SEIFA for Sydney metropolitan area is 1067. Preschools which had been established to provide services for disadvantaged, low income families were specifically targeted to recruit participants from a low SES background (median SEIFA = 891).

Families from a Chinese background were recruited through a community health centre in a region with a high Chinese population and were all from high SES backgrounds (median SEIFA = 1100). Participants from a Middle-Eastern background were accessed using the children's service agency as well as a privately run early childhood facility in a region with a high Middle-Eastern population (median SEIFA = 914).

Six centres were invited to participate in the study and five centres agreed (4 preschools and 1 community health centre). One preschool centre declined because of staff shortages. Nine focus groups were conducted involving a total of 39 participants (22 parents, 17 preschool staff). Table 1 describes the participant characteristics. The number of participants was determined by the achievement of data saturation. That is, no new findings were identified with the numbers listed above.

**Table 1:** Participant characteristics

**Parents**		**n = 22**
Mothers		20
Fathers		2
***Target groups***:	***SEIFA Index*^**1**^** **(median)**	
1. Low socioeconomic	891	10
2. Middle-Eastern	914	7
- Lebanon (6)		
- Syria (1)		
3. Chinese	1101	5
- Hong Kong (2)		
- Malaysia (1)		
- China (2)		

**Preschool staff**^**2**^		**n = 17**

Female		17
- Directors		3
- Trained teachers		5
- Assistant staff		9
***Years in employed in childcare sector:***		
≥ 20 years		5
10–19 years		4
5–9 years		5
< 5 years		3

^1^SEIFA Index of Relative Socioeconomic Disadvantage median. (Sydney median: 1067).

^2^varied ethnicities including: Anglo-Celtic, Mediterranean, Middle-Eastern, Chinese

### Procedure

Focus groups were conducted between November 2006 and December 2007. All focus groups were held at the participating centres and ran separately for parents and staff. Each session was conducted over 60–120 minutes and was facilitated by one health professional and the first author (GMD). All focus groups were recorded and transcribed for analysis purposes.

The sessions were semi-structured, commencing with a series of open-ended questions which were designed to stimulate discussion about physical activity in young children (see Table 2) but the sessions were flexible to optimize the natural flow of ideas and conversation in the group. The major topic areas included the nature, value, and patterns of physical activity in young children, facilitators and barriers to physical activity, and recommendations about physical activity and SSR.

**Table 2:** Focus group schedule of questions

**Topic area**	**Questions**
Participants' understanding of nature of physical activity	• When I say 'physical activity', what does it mean to you?
	• What words would you use to describe the difference in physical activity intensity in activities that young children do? What words would you use to describe the difference between a child who moves slowly and a child who always active and on the go?
	• What is the value of physical activity for young kids? Older children? Adults?
	• Tell me your thoughts about physical activity and your child's health.

Pattern of physical activity	• Where does physical activity fit in your child's day?/How does physical activity fit into the child's day at this centre?
	• Do you see differences in level of physical activity between boys and girls who attend this Centre?

Influences upon physical activity behaviour	• What makes your child/children active?
	• How do you encourage your child to be active?/When you spend time with your child what do you like to do together?
	• How do you as staff agree as to what to promote for children attending this Centre?
	• Do you or your partner do different things with your child?
	• How does being at preschool influence your child's physical activity?
	• Do your children spend time with extended family such as aunts/uncles/cousins/grandparents? How does this contact influence how active your child is?
	• Does anything make it difficult for them to be active?

Pattern of small screen recreation	• Can you tell me about your child and their usual TV routine, use of computers or things like that?
	• What influences this?

Knowledge of recommendations/guidelines about physical activity and SSR	• Do you know of any guidelines about what is recommended for children in regards to physical activity, TV viewing and use of computers?

Note: The sequence of questions varied during each focus group.

The study was approved by the Ethics Committees of The Children's Hospital at Westmead and The University of Sydney. Informed consent was obtained from all participants and the anonymity and confidentiality of the participants was ensured.

### Data analysis

Thematic analysis of the focus group audio files, written transcripts and detailed field-notes, was an iterative process that took place during and after the period of data collection. A framework to code the data was based upon the study aims, the question schedule and the major topic areas. Significant ideas and themes were coded and reviewed by three members of the research team (GMD, LAB and JH); this process continued until a point when no new findings were identified from the texts (data saturation). Review of emergent themes was conducted by the three members of the research team who agreed upon the final list of themes.

## Results

### Description of physical activity

Participants described physical activity as any form of body movement and they recognised that it extended beyond large body movements. Several participants also noted the converse of being physically active; in particular, many stated that it was not sitting in front of the television. Statements that typified participants' description of physical activity include:

"... as long as they're sort of active, they're moving ... yeah as long as they're moving." (Parent, low SES group)

"Not being in front of the television ... I think anything to do with moving." (Parent, Middle-Eastern group)

Descriptions of unstructured physical activity in young children included walking, running, jumping, climbing, chasing games, ball games, using play equipment (for example, swings or trampolines), bike-riding and dancing. The majority of parents (73%) had involved their children in organised activities such as swimming lessons, gym programs that focussed on the development of motor skills, dancing classes and/or movement and music classes. Only a small number (13.6%) of parents had enrolled their children into formal sporting or recreational activities, but, most parents reported that they were considering these options when their children were of school age.

Parents and staff recognized the contrast in activity behaviour between young children, older children and adults. Specifically, they described young children's activity as typically sporadic and short duration:

"... little kids, they just don't stop. She grabs her rabbit, plays around with that and then she drops that and goes and gets the guinea pig ... and then the trampoline, the swings ... anything." "... because that's what they're doing basically all day isn't it? – running around, doing something and they don't sit down much. They're up again, and back and forward." (Parent, low SES group)

In addition, participants considered the term 'intensity', (i.e., light, moderate, vigorous) which is used to describe older children and adult physical activity participation was not applicable to young children's physical activity patterns. Instead they saw activity as polar opposites, (e.g. high versus low, on versus off).

" ... I don't think anyone's actually in the middle, they're either fast or they're not. They might play a puzzle and that's the dawdling part and the vigorously is when they're off and running. I don't think there's anything in between." (Parent, low SES group)

### Value of physical activity

Parents and staff identified the benefits of children being active, including (a) health benefits (increased muscle and bone development, 'brain development', motor skill development, increased metabolism and the prevention of obesity), (b) psychological benefits ('energy release' resulting in a more settled behavioural state, mental stimulation, and an increased sense of happiness and well-being), and (c) social benefits (developing relationships with peers and adults and learning important social skills such as turn taking).

### Influences upon physical activity

A summary of influences reported by study participants is provided in Table 3.

**Table 3:** Influences on physical activity: key themes and concepts from focus groups

**Concept**	**Themes**
**Facilitators**	• Child's preference for activity
	• Positive parental, sibling or peer modelling
	• Access to safe play areas, organised activities and preschool or day care programs
	• Sense of social connectedness
**Barriers**	• Safety concerns (child and neighbourhood)
	• Time constraints
	• Financial restraints (lower socioeconomic)
	• Cultural values towards educational achievement (Middle-Eastern)
	• Regulations about equipment and sun exposure
**Parents**	The inherent nature of young children is to be active and television is the biggest barrier to children being active.
**Preschool staff**	The art of creative, active play needs to be restored in the lives of young children

#### (a) Facilitators of physical activity

Facilitators of physical activity included a child's preference for activity, positive parental modelling, sibling and/or peer modelling, access to safe play areas, organised activities and preschool or day care programs and a sense of social connectedness. Participants recognised that children have different personality traits and that some children are more inclined to be active than others. Parents also considered that children in the preschool-age group generally had an inherent tendency to be active compared with older children.

Preschool staff suggested that personality was only one driver of a child's physical activity and that the social and physical environments played a key role which encouraged physical activity independent of the child's personality traits. 'Scaffolding' was used to promote activity which included (a) creating play environments that built upon an individual child's interests to extend their activity behaviour, (b) adult modelling and encouragement and (c) peer behaviour

"It's your environment ... that you have set up. Materials, joining in with them, helping them ... helping them to make social links, scaffolding. We always talk about 'scaffolding' play in childhood now ... the adult becomes the playmate to help a child to become involved and participate ... I think it goes with the set up too, have the things available and ready to go. The yard looks inviting and interesting ... safe. The inspiration for the children to be involved comes from what they see. When they see something that looks really interesting then they just naturally want to do it ... (and) where you position yourself... staff and adults are inviting to children as well. So, positioning the staff also encourages the children to be involved in those particular activities." (Preschool staff member)

The importance of parental modelling and/or encouragement of physical activity was emphasised by parents in this study as being a key influence of physical activity and sedentary behaviour in children.

"I think that it all comes back down to the parents because if they let them sit there and do nothing, well of course they're just going to sit there and do nothing. ... I am an outside person and so is my husband ... we're not telly people. So I guess that that sort of has rubbed off on my kids." (Parent, low SES group)

In addition to parental modelling, parents also believed that active siblings, a sense of social connectedness (i.e., extended family networks, neighbourhood communities), access to safe play areas and involvement in preschool or day care programs, were other positive influences on physical activity behaviour.

While the parents involved in our study uniformly supported the encouragement or modelling of activity, preschool staff commented that such behaviour was not consistently evident among parents. Many of the children in the low SES preschools were from single parent families and staff suggested that stresses associated with social disadvantage could adversely influence parent-child interactions in terms of playing and that these parents had limited time and financial resources to involve their child in organized activities.

Another factor identified by preschool staff catering for Middle-Eastern communities was the potential for both cultural and language barriers to exacerbate a perceived lack of appreciation of the importance of physical activity, which was seen as indifference towards the need for physical activity.

" ... we have a lot of mothers that drop off their kids early in the morning ... and they pick them up late ... they go on to coffee with their friends, do shopping, clean their house, cook for their husbands ... the minority of parents want to be involved or want to know what is happening with their children but the majority don't care ... there is a language barrier for some ... so I think (the) language barrier plays a big role ... for parents being withdrawn out of their children's lives." (Preschool staff member)

#### (b) Barriers to physical activity

Barriers to physical activity included parental safety concerns exacerbated by negative media stories, time restraints, financial constraints (in the low SES group), cultural values favouring educational achievement (in the Middle-Eastern group), and among preschool staff, safety regulations about equipment design and use within the preschool environment.

The majority of parents were concerned about their child's safety (81%) at both a personal and community level. Many parents acknowledged a fear of allowing their children to engage in activities that tested the child's physical limits (e.g., when using playground equipment) for fear of the child sustaining injury.

"I'm like if they're on the swings, 'oh, be careful, be careful' ..." (Parent, Middle-Eastern group)

Preschool staff also noted a negative impact of overprotective behaviour in parents, as exemplified in the comment below:

Sometimes parents of young children might be very protective and they might not let them venture and try out those things out ... and they (the children) are like 'I don't want to do it; I don't want to hurt myself.' (Preschool staff member)

Parents of Chinese ethnicity expressed the belief that being overprotective was a cultural trait. These participants explained that they had made conscious effort to counteract this attitude because of (a) the recognition of the importance of physical activity and (b) the value of setting positive habits early in a child's life:

"But I think sometimes that it is our background as Asians. I think they tend to overprotect our kids. I can see that in the park you know you ... can see the difference between the Australian and the Asians ... I think I am more lenient ... you know encouraging more physical activity especially when you are in school is important. The three to five year old, if you promote that at that age it becomes built into their lives ... they do not think twice about doing it, they just go and do it and I think that's important rather than saying you have to make a conscious effort to say 'I need to exercise', it's part of your lifestyle. You just do it." (Parent, SE Asian group)

Excessive road traffic near residential areas or public parks was a significant neighbourhood safety concern of most participants. As a consequence many parents restricted outdoor play for their children to backyard areas. When or whether to allow their children (when older) to walk around their local neighbourhood or to allow them to walk to school was a future concern expressed by several parents. Parents and staff acknowledged that walking was a healthy activity but the perceived threat of injury was a barrier to active transport.

An exception to community safety concerns was noted by parents who detailed strong social connectedness either through extended family networks who lived in close proximity, or close social relationships in the immediate neighbourhood. In this instance, parents were confident in extending their children's outdoor play boundaries beyond the immediate home yard as highlighted by the following comment:

"Like we have got so many cousins and family friends that live in the one street or like a block away and they know who your child is. So (it's) more protective, you have got a watchdog out there for you." (Parent, Middle-Eastern group)

Safety concerns were reflected in the manner in which preschool staff accommodated statutory public liability and regulation policies. Staff commented that change in regulations was exemplified in current equipment design (e.g., height restrictions in climbing apparatus). They considered this change had altered play experiences for children so that they were not always being challenged to their physical limits. While children could still be active at preschool, some staff expressed concern that children were not learning how to deal with play situations in public parks, such as in climbing and use of swings. Staff felt this situation could further contribute to over-protectiveness by parents.

A few staff also suggested that safety concerns were a factor that may explain an emerging pattern of over-timetabling children into 'packaged play'. That is, instead of being left to create their own games outdoors, staff believed some children were being enrolled in organised activities to be under adult supervision and therefore safe.

As noted above, the majority of parents elected to enrol their children in organised activities to enhance their child's overall health and activity experiences. However, there was varied experience of organised activities. In low SES communities financial constraints inhibited what families could access for their children. Staff and parents from the two centres catering for children from Middle-Eastern backgrounds gave contrasting observations about children and organised activities. Few children from the centre in which staff considered parents had an indifferent attitude, were involved in organised physical activities. The staff considered that these parents displayed high educational expectations for their children.

"Well my parents they do have this thing in their head where they want their children to get ready for school and then they see what children do throughout the day and they go 'how come they're always playing?' I really, really need to educate my parents where they see play as a learning experience ..." (Preschool staff member)

Staff at the other centre catering for children from a Middle-Eastern background felt there was a tendency by parents to over-timetabling children's activities. Activities included enrolment in religious and language coaching to assist with school entrance exams. Staff expressed concern that this routine inhibited free play time.

"... education pressure it's really major ... some parents think it is absolutely ridiculous but they have to do it as they want their children to go to these schools, whereas others think it's wonderful and you have got to train them (the parents) up basically ... make sure they're (the children) not involved in too many organised activities after preschool because for lots of children their whole life is a timetable ... I just say that it is really good for them to have free time just to play ... they do need some time to relax and just be still as well, not racing from one appointment to another." (Preschool staff member)

Some staff observed gender differences in parental encouragement of activity. Specifically, staff perceived that many girls from Chinese or Middle-Eastern backgrounds were dressed in a manner that inhibited active play or parents openly discouraged girls from being active and getting dirty. In contrast, the parents expressed a determination to provide children of both sexes with equal play experiences. A few parents acknowledged that different gender expectations were sometimes imposed by older generations and this situation created potential conflict within extended families units.

### Small screen recreation (SSR)

Television and DVD viewing were the main form of SSR reported by parents although many stated that their children used video games and computers regularly. The attraction to these forms of SSR reflected behaviours modeled by either parents or older siblings. SRR was accepted by the parents as being an inevitable consequence of the current technological era which provided educational opportunities, but many parents were concerned at the effect of excessive SSR.

"Sitting down in front of the TV ... their brains are not working ... they're like zombies ... if my son's on the Playstation^® ^he becomes aggressive ... he's a totally different person ... if there is a Playstation^® ^it really distracts him from doing other stuff." (Parent, Middle-Eastern group)

Television viewing habits fell into several patterns. Some parents permitted television viewing early in the morning to occupy their children while preparing for the day's routines (e.g., when the parent was showering and dressing). Other parents discouraged morning television viewing as they felt that it distracted children from completing their own morning routines (such as dressing and breakfast). Television was occasionally used as a 'babysitter' to allow longer sleep-ins for parents. A favoured time for television viewing was after preschool. Parents generally restricted viewing to 1–1.5 hours but in wet weather, television and DVD viewing time increased. This approach was adopted to keep children occupied without the need for constant parental attention.

Parents stressed that excessive television viewing was the greatest barrier to young children being active. The majority of parents in this study consciously moderated their children's television viewing and other SSR. Otherwise they felt their children would spend several hours engaged in these activities.

### Knowledge of physical activity and small screen recreation guidelines

Only three participants (7.7%) were familiar with national guidelines about SSR and none were familiar with physical activity guidelines for any age group. Preschool staff adhered to guidelines from (a) their State Department of Sport and Recreation which promoted that programs should include a balance of time spent indoors and outdoors [23] and (b) NSW Cancer Council SunSmart policy guidelines regarding time spent outdoors during summer months [24]. Parents used personal judgement to moderate or discontinue children's SSR by assessing if their child had a 'zombie look' or if they displayed excessive aggressive behaviour

### Key concepts about young children, physical activity and play

The significant concepts which emerged from this study are listed in Table 3. Firstly, parents perceived young children were inherently 'programmed' to be active and that over time society 'de-programs' them by exposing children to activities that are appealing but increasingly sedentary, such as SSR. Parents were primarily concerned about television and DVD viewing however there were increasing concerns about exposure to other technologies such as computers and hand-held screen games (e.g. Gameboy^®^, Tamagotchi^®^) which was occurring at increasingly younger ages. Parents strongly expressed the belief that if they could control the time spent on these activities then children would naturally be active.

Secondly, staff were concerned about SSR and the effects of increased exposure to video or computer games on the nature of children's play. Many believed that the art of active, creative play was being lost in the current generation of young children and they felt that if children are simply left to their own devices they become bored and defer to the instant gratification of SSR. Additionally, the over-timetabling of some children into extracurricular, supervised or 'packaged' play also increased the risk of children not being adept at creating their own play activities. Staff felt that the art of creative play needed to be restored to the lives of young children.

## Discussion

The findings from this study support the relevance of applying the socioecological model of behavioural influences to examine young children's physical activity. The identified influences upon young children's physical activity behaviour included (a) personality traits within the child; (b) functioning within the family unit including parental attitudes and capacities, and modeled behaviours by parents, siblings and peers; (c) attitudes, policies and regulations within preschool facilities; (d) social connectedness within the broader community; (e) perceived safety of the neighbourhood environment and (f) access to areas and facilities that promote physical activity. Similar influences at these varying levels have been reported in older children and adults [2,25-33].

Compared with older children and adults, there are fewer studies that have specifically examined influences upon physical activity in young children [17,34-42] and very few that have utilised a qualitative research approach [43-46].

The participants in this study had a clear insight into the nature and value of physical activity. An important finding of this study was a strong sentiment that the term 'intensity' was not applicable to young children's physical activity patterns. Current physical activity guidelines for children aged 5–18 years and adults, are framed according to duration, intensity and frequency of activity [47-50]. A different framework or lexicon may be required for young children if activity guidelines are to be meaningful to parents and carers of young children.

The types of physical activity reported by the participants were very similar to studies involving Hispanic, Canadian and Australian preschool-age children [45,46,51]. This suggests that young children engage in similar physical activities within varying ethnic groups across developed countries.

Although participants were aware of the benefits of physical activity, none was familiar with any formal guidelines about the amount of physical activity that should be encouraged in children. Preschool staff adhered to a very broad recommendation provided by their State Department of Sport and Recreation [23], and they complemented this with safety recommendations related to time spent outdoors provided by the NSW Cancer Council SunSmart policy [24]. As more specific recommendations about optimal physical activity behaviours in young children emerge, it is important that these recommendations are communicated effectively. More precise regulations would also assist to ensure that all child care agencies are promoting appropriate activity programs. A US study showed physical activity program standards varied amongst agencies with an impact upon activity behaviours in the children attending those centres [52].

In this age group, the influence of parents upon child's behaviour is critical [19]. Our participants especially acknowledged that parental modeling and/or encouragement of activity was a key influence and predictor of physical activity and sedentary behaviour in children. Parental physical activity has been recognised as a correlate of physical activity among preschool children [17,42].

Television viewing and other SSR has been identified as a negative influence on children's physical activity [9]. It was seen as an important parental role to moderate television viewing habits, as well as other SSR. In our study only three participants (7.7%) were familiar with the national guideline for SSR which recommends children aged 2–18 spend no more than 2 hours/day on quality viewing [47,53]. Instead parents used personal judgement to gauge the amount of time their children used SSR and this usually related to undesired behavioural changes noted in their child such as a 'zombie look' or display of aggressive behaviour. Our participants, especially the parents, stressed that in the young age group television viewing was the greatest barrier to children being active. A strong opinion expressed by parents was that if the television was turned off then children in this age group would naturally be active. Further investigation of this hypothesis would be helpful to guide possible health promotion interventions.

Parental attitudes and behaviours frequently reflect broader cultural issues and beliefs which may give rise to competing agendas, for example a focus upon education at the expense of participation in physical activity. The importance of educational achievement has previously been reported among Chinese cultures [54-56] but is less well publicised in Middle-Eastern communities, although for Lebanese families in Australia the value of education is considered an important means of upward social mobility [57]. These issues and challenges were raised by both parents and preschool staff who catered for children from a Middle-Eastern background. The degree of focus upon educational achievement in this group was an unexpected finding of this study.

Safety was the major issue of concern, and barrier to physical activity, reported by participants. Safety concerns were identified at two levels; firstly at the child's personal level with parents acknowledging a tendency by some to over-protectiveness and secondly, at a broader community level with concern about predators and traffic. The concept of over-protectiveness or the "bubble-wrap" phenomena has been noted previously [58]. These findings about broader neighbourhood safety issues align with studies in older children [18,59,60]. Social connectedness would appear to be a potential counter to these neighbourhood issues.

A potential consequence of safety concerns, along with the infiltration of technology into the lives of children, is a change in the nature of young children's play. Staff in our study commented on this change. Specifically they felt that the art of creative play needed to be restored to the lives of young children: a concept which has been advocated by others [4,53]. In a recently published consensus paper, the American Academy of Pediatrics explored the issue of reduced child-driven play and its potential repercussions. The Academy advocate that free play should be included along with academic and social-enrichment opportunities and that safe environments be made available to all children [[53] page 188]. Achievement of this ideal may be some time off but this approach does create the potential for healthy activity habits to be established early in life.

### Limitations

Selection bias may be considered a limitation of this study because all participants had a keen interest in the area of physical activity in young children. Additionally, only a limited number of participants in the study were male (2/39) and because the focus groups were conducted during the day it limited the participation of parents who were employed in full-time work.

## Conclusion

The findings support the relevance of the socioecological model of behavioural influences to young children's physical activity. In addition, the results of this study suggest that in the preschool-age group, efforts to promote and to establish positive habits towards physical activity may best be directed at (a) emphasising national guidelines for SSR in order to reduce time spent in this behaviour (b) educating families and carers about the importance of creative, free play to reinforce the child's inherent nature to be active and (c) taking initiatives to develop social connectedness in order to create safe play environments for children.

## Competing interests

The authors declare that they have no competing interests.

## Authors' contributions

GMD conducted the study and drafted the paper. JH was involved in the design of the study, assisted with thematic analyses and contributed to the drafting of the paper. LAB was involved in the design of the study, assisted with thematic analyses and contributed to the drafting of the paper. LLH contributed to the drafting of the paper. All authors read and approved the final manuscript.
